# Invasive anisakiasis by the parasite *Anisakis pegreffii* (Nematoda: Anisakidae): diagnosis by real-time PCR hydrolysis probe system and immunoblotting assay

**DOI:** 10.1186/s12879-017-2633-0

**Published:** 2017-08-01

**Authors:** Simonetta Mattiucci, Michela Paoletti, Alessandra Colantoni, Antonella Carbone, Raffaele Gaeta, Agnese Proietti, Stefano Frattaroli, Paolo Fazii, Fabrizio Bruschi, Giuseppe Nascetti

**Affiliations:** 1 Department of Public Health and Infectious Diseases, Section of Parasitology, “Sapienza University of Rome” and “Umberto I” Teaching Hospital, P.le Aldo Moro, 5, 00185 Rome, Italy; 2 Department of Ecological and Biological Sciences, “Tuscia University”, Largo dell‘Università s/n, 01100 Viterbo, Italy; 3 Department of Surgical Sciences, “Sapienza - University of Rome” and “Umberto I” Teaching Hospital, Rome, Italy; 4 Department of Translational Research, N.T.M.S., Pisa University, Pisa, Italy; 5 U.O. Pathological Anatomy III, Department of Surgical, Medical and Molecular Pathology and Clinical Care Medicine, University of Pisa, Pisa, Italy; 6 “S. Spirito” Hospital, Pescara, Italy

**Keywords:** Anisakiasis, Italy, *A. pegreffii*, Molecular diagnosis, Real-time PCR, Serodiagnosis, Immunoblotting

## Abstract

**Background:**

Anisakiasis is a fish-borne zoonosis caused by *Anisakis* spp. larvae. One challenging issue in the diagnosis of anisakiasis is the molecular detection of the etiological agent even at very low quantity, such as in gastric or intestinal biopsy and granulomas. Aims of this study were: *1*) to identify three new cases of invasive anisakiasis, by a species-specific Real-time PCR probe assay; *2)* to detect immune response of the patients against the pathogen.

**Methods:**

Parasite DNA was extracted from parasites removed in the three patients. The identification of larvae removed at gastric and intestinal level from two patients was first obtained by sequence analysis of mtDNA *cox2* and EF1 α-1 of nDNA genes. This was not possible in the third patient, because of the very low DNA quantity obtained from a single one histological section of a surgically removed granuloma. Real-time PCR species-specific hydrolysis probe system, based on mtDNA *cox2* gene, was performed on parasites tissue of the three cases. IgE, IgG_4_ and IgG immune response against antigens *A. pegreffii* by Immunoblotting assay was also studied.

**Results:**

According to the mtDNA *cox2* and the EF1 α − 1 nDNA sequence analysis, the larvae from stomach and intestine of two patients were assigned to *A. pegreffii.* The Real-time PCR primers/probe system, showed a fluorescent signal at 510 nm for *A. pegreffii*, in all the three cases*.*

In Immunoblotting assay, patient CC1 showed IgE, IgG_4_ reactivity against *Ani s 13*-like and *Ani s 7*-like; patient CC2 revealed only IgG reactivity against *Ani s 13*-like and *Ani s 7*-like; while, the third patient showed IgE and IgG reactivity against *Ani s 13*-like*, Ani s 7*-like and *Ani s 1*-like*.*

**Conclusion:**

The Real-time PCR assay, a more sensitive method than direct DNA sequencing for the accurate and rapid identification of etiological agent of human anisakiasis, was successfully assessed for the first time. The study also highlights the importance to use both molecular and immunological tools in the diagnosis of human anisakiasis, in order to increase our knowledge about the pathological findings and immune response related to the infection by zoonotic species of the genus *Anisakis*.

## Background

The larval parasites of the genus *Anisakis* are considered the most important biological hazards present in “seafood” products. The accidental ingestion of third stage larvae (L3) of *Anisakis* spp. by consuming infested raw, undercooked, or improperly processed (e.g. marinated) seafood, causes a parasitic zoonosis, known as anisakiasis. The larvae do not mature in the human host; however, they are implicated in two forms of anisakiasis: “not invasive” and “invasive”. The “not invasive” form occurs when the larva is not able to penetrate the gastric or intestinal mucosa and it remains luminal; in this case, the patient could be asymptomatic and the larva could be eliminated with stools and/or vomit. The “invasive” form is characterized by different steps of the parasite invasion: *i)* attachment to, *ii)* embed into, and finally, *iii)* the penetration by the *Anisakis* sp. larva in the tissues, usually the gastric and intestinal mucosa. In the latter case, severe gastrointestinal symptoms during the acute phase of infection, such as epigastralgia, nausea, vomiting, abdominal pain are described. The chronic infection of anisakiasis, is characterized by the *Anisakis* larvae deeply invading the gastric and intestinal walls, causing direct tissue damage, ulcers and, eventually, eosinophilic granulomas [1–5].

In addition, gastrointestinal anisakiasis may be accompanied by IgE-mediated allergic reactions, ranging from urticaria or angioedema to anaphylaxis, associated to the active penetration of live larvae into the gastric mucosa [3].

Currently, nine species are genetically recognized in the genus *Anisakis*, among which two (i.e. *A. simplex (s. s.)* and *A. pegreffii*) are relevant for humans, mainly because of their zoonotic role as etiological agents of anisakiasis [6]. *A. pegreffii* is the most widespread anisakid species affecting commercial fish from Mediterranean waters; its geographical distribution includes the Iberian Atlantic coast waters, as well as the Atlantic and Pacific Austral waters [7, 8]. Indeed, so far *A. pegreffii* is reported as causative agent of invasive anisakiasis in Europe (Italy, Croatia) [9–13], but also in Japan [14, 15] and South Korea [16]. It has been demonstrated with molecular markers that *A. pegreffii* is able to cause gastric [9, 10, 12, 14–16], intestinal [11, 13] anisakiasis and gastroallergic anisakiasis [12].

One of the most challenging issue in the diagnosis of anisakiasis in humans is the possible detection of the DNA of the etiological agent even when present in very low quantity, such as it could occur in bioptic tissues and histological sections obtained from eosinophilic granulomas that were surgically removed. In these cases, the possibility to use a diagnostic tool based on a molecular technique specific, rapid and of high sensitivity, is crucial for the diagnosis of this foodborne pathogen. In this context, the Real-time PCR (RT-PCR) has overcome some limitations related to the DNA quantity and the time, with respect to the conventional PCR-DNA direct sequencing of target genes [17].

Here, we describe three new cases of invasive anisakiasis based on direct DNA sequencing of some target genes, and by a species-specific RT-PCR hydrolysis probe assay, recently developed for species of the genera *Anisakis* and *Pseudoterranova,* based on the mtDNA *cox2* gene [unpublished data]. In addition, immune response in the three patients by Immunoblotting assay against antigens/allergens *A. pegreffii* was performed.

## Cases presentation

**Fig. 1:**
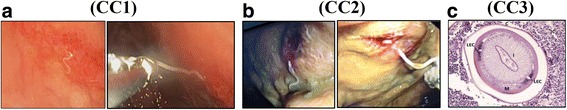
**a** CC1: the larval nematodes were removed by using biopsy forceps from the gastric mucosa; **b** CC2 the larval nematode was retrieved by forceps from the lumen of the intestine; **c** CC3: histological section of the surgically removed eosinophilic granuloma in which a nematode in a cross section was visible (H&E stained, 100X). C = cuticle; M = muscular layer; I = intestine; LEC = Y-shaped lateral epidermal chords; IC = inflammatory cells

First clinical case (here by indicated as CC1): a woman was admitted to the Emergency Department of the “Santo Spirito Hospital” in Pescara, Abruzzo Region, Italy, complaining of epigastric pain and nausea after eating raw marinated anchovies. The patient was not suffering of urticaria. At the endoscopy, a marked mucosal swelling was found, with two worms found around the area, penetrating the gastric mucosa. The worms were extracted from the gastric mucosa by means of biopsy forceps (Fig. 1a). After extraction, the pain subsided within a few hours. The worms were then fixed in alcohol for further molecular identification.

Second clinical case (hereby indicated as CC2): a 58-year-old man was hospitalized at the Emergency Department of the “Umberto I Teaching Hospital” in Rome, Latium Region, Italy. The patient referred a sense of oppression in right side of the abdomen. At the anamnestic history, the patient referred that a week before, he had eaten raw marinated anchovies, and 2 days after consumption the symptoms appeared. No urticaria or angioedema occurred. A colonoscopy was carried out. During the colonoscopy, at the physical examination, tenderness without rebound tenderness in the right iliac fossa was revealed; splenomegaly or palpable mass in the abdominal area was not observed. The colonoscopy was performed up to the caecum tract. It showed an area of approximately 2.5 cm centro-lateral to the ileo-caecal valve where the mucosa appeared hyperemic with the presence of two erosions covered by fibrin. While operating for bioptic tissue around that area, the presence of a live worm (about 2 cm in length) was detected (Fig. 1b). It was then removed with biopsy forceps, and stored in alcohol until its molecular identification. Symptoms quickly resolved after endoscopic removal of the larva. Six months later a colonoscopy was performed again, resulting negative.

Third clinical case (hereby indicated as CC3): a 35-year-old man, who was hospitalized at the “Surgical Emergency Unit” of the Leghorn Hospital, Tuscany Region, Italy. The patient presented abdominal pain, nausea, and fever caused by an acute intestinal obstruction. At the anamnestic history, the patient referred to have eaten raw anchovies several days (10 days) prior to admission in the hospital. A laparoscopic right hemicolectomy and a bowel anastomosis were then performed. Macroscopically, the ileum and the appendix did not show any alteration, while the colon presented a large polypoid circumferential neoformation with smooth surface of 5.5×5×3 cm that was obstructing the lumen of the colon 5.5 cm far from the distal margin. The surgical specimen consisted of a tract of the small bowel; the ileo-caecal valve was stenotic and the mucosa appeared to be extensively edematous. The tissue sample from the organ showing the lesion was fixed in 10% neutral buffered formalin, embedded in paraffin, sectioned at 6 μm and stained with haematoxylin and eosin.

Written informed consent was obtained from the patients for the publication of the cases reports.

## Methods

### DNA extraction

Total genomic DNA was extracted from 2 mg of homogenized tissue from the two nematodes removed by endoscopy in CC1 and from one larva of CC2, by using the “DNeasy blood and tissue” kit (Qiagen), following the manufacture’s protocol.

In CC3, a single one histological section, obtained from the surgically removed granuloma at the intestinal level, was available. Thus, the larval nematode - embedded in the paraffin - was gently removed by scratching the histological slice from the glass-slide, by using a glass rod bearing a needle tip. After that, the parasite genomic DNA extraction was carried out according to procedures previously described [11].

The genomic DNA obtained from those samples are stored at the research laboratory of S. Mattiucci, Department of Public Health and Infectious Diseases (Section of Parasitology) - ‘Sapienza University of Rome’.

### PCR condition and DNA sequencing

In CC1 and CC2, the identification at species level was previously obtained by direct sequences analysis of mitochondrial (mtDNA *cox2,* 629 bp) [18] and nuclear (elongation factor EF1 α-1 of nDNA, 409 bp) genes [19]. Whereas, the extremely low amount of DNA (<0.01 ng/μl, Qubit2.0 BR-dDNA kit) obtained from the single one histological slice available in CC3, did not allow its direct sequencing.

The mitochondrial cytochrome c oxidase subunit II (*cox2*) gene was amplified using the primers 211F (5′-TTTTCTAGTTATATAGATTGRTTYAT-3′) and 210R (5′-CACCAACTCTTAAAATTA TC-3′) [17]. Polymerase chain reaction (PCR) was carried out according to the procedures as previously described [18]. The sequences obtained at the mtDNA *cox2* for the three larval nematodes, analyzed in the present study, were compared with those already obtained for the same gene in the species *A*. *pegreffii* [18].

The elongation factor (EF1 α − 1 nDNA) nuclear gene was amplified using the primers EF-F (5′-TCCTCAAGCGTTGTTATCTGTT-3′) and EF-R (5′-AGTTTTGCCACTAGCGGTTCC-3′) according to Mattiucci et al. [19]. The PCR conditions and procedures followed those reported in Mattiucci et al., 2016. The sequences obtained at the EF1 α − 1 gene of the nDNA for the three larval specimens and analyzed in the present study were compared with those already obtained for the same gene in the species *A*. *pegreffii*, as previously described [19]*.*

### RT-PCR probe assay

The identification at species level of CC3 was obtained by using a RT-PCR species-specific primers/probe system [see below] based on mtDNA *cox2* gene. The same method was applied in the DNA obtained from the larvae of CC1 and CC2. In addition, in order to verify the specificity of the RT-PCR primers/probe system and to test a possible cross-reactivity with the host tissue, the genomic DNA from human blood (*h*) was also tested in the same reaction. The RT-PCR primers/probe system was carried out using the primers and specific probe for *A. pegreffii* as follows: forward primer RTpegF-CTTTTGGAGGTTGATAATCG and reverse primer RTpegR-CCCACAAATCTCTGAACATT; species-specific probe pegHyPr-CTTGGGCTTTGCCTAGGATGTC (dual labeled with 6FAM/BHQ) [our unpublished data]. PCR amplification reaction was performed in a total volume of 15 μl, containing 7.5 μl of LC FS DNA HyProbe MASTER (2×) (Roche®), 0.3 μl of primers (0.2 μM), 0.75 μl of probe (0.3 μM) and 1.5 μl (3–5 ng/μl) of DNA template. Reactions were carried out as follows: 95 °C for 10 min (initial denaturation), followed by 40 cycles at 95 °C for 10 s (denaturation), 55–65 °C for 30 s (annealing), 72 °C for 1 s (extension), followed by a cooling step at 40 °C for 30 s. The reactions were run in a Light Cycler 480 II System (Roche®) Real Time PCR instrument, using LightCycler 480 Multiwell Plate-96 white (Roche®) and LightCycler 480 Sealing Foil (Roche®). Samples were tested in duplicate. The results were analyzed using the LC480 dedicated Software.

The parameters of efficiency and detection limit were estimated to test the performance of the assay. The efficiency of primers was tested; an amplification reaction was considered optimal if the efficiency value was 2 (*E* = 10^(−1/slope)^); the *E* value was automatically calculated by the software program of the RT- Roche Instrument), because the amount of target DNA would double with each amplification cycle [20]. The *Slope* of the standard curve describes the kinetics of the PCR amplification and indicates how quickly the amount of target DNA can be expected to increase with the amplification cycles; in an optimal amplification reaction, the *slope* value was −3.3, in relation to the *E* value.

In addition, in order to establish the minimum amount of DNA detectable by the system, the Limit Of Detection (LOD) was established, preparing 16 serial 2-fold DNA dilutions starting from a known amount of DNA (from 20 ng/μl to 0.0006 ng/μl).

### Immunoblotting assay

The serum samples for the serodiagnosis for *Anisakis* on those patients analysed by Immunoblotting assay were obtained from CC1 just during its recovery, from CC2 after 1 week, and finally from CC3 1 week after the surgical removal of the granuloma. Sera samples are stored at the Section of Parasitology, Department of Public Health and Infectious Diseases of “Sapienza - University of Rome” (First Author’s Laboratory).

The immune response was tested against antigens/allergens *Anisakis pegreffii* larvae for: IgE and IgG_4_ in CC1, IgE and IgG in CC2 and CC3. In particular, Excretory/Secretory (ES) antigens obtained from in vitro culture of *A. pegreffii*, were used in the Immunoblotting assay [21]; they followed the designation as reported in our previous paper [21].

Antigens from crude extracts and ES were run through protein gel electrophoresis. Proteins were diluted with dissociation buffer (1:1) and boiled at 95 °C for 5 min. Boiled proteins were run through 12% SDS-polyacrylamide gel and transferred on a nitrocellulose membrane by Mini-PROTEAN 3 Cell (Bio-Rad).

Membranes were incubated overnight at 4 °C with patient’s serum (IgE 1:20 dilution; IgG 1:35; IgG_4_ 1:20). After three washes, the membranes were incubated with alkaline phosphatase-labelled monoclonal anti-human IgE (Sigma-Aldrich) at a 1:2000 dilution, IgG (Sigma-Aldrich) at a 1:2500 dilution and IgG_4_ (Sigma-Aldrich) at a 1:1000 dilution. Finally, band detection was carried out with 5-bromo-4-chloro-3-indolyl phosphate/nitro blue tetrazolium (Sigma-Aldrich) for 20 min.

## Results

### Histological analysis of CC3

Histologically, the neoformation was composed by colic wall with marked edema of the submucosa and muscular layer with abundant transmural inflammatory infiltrate composed mainly by lymphocytes, plasma cells and over all eosinophils. The histological examination of the eosinophilic granuloma, of the overall size of about 0.15 cm, revealed the presence of a parasitic nematode, having a diameter of 0.48 × 0.30 mm, with a thin cuticle lacking of lateral alae. Polymiarian muscle cells, separated into four quadrants by the chords, having two wing-like distal lobes, were well visible; the nematode’s intestine was circular with a triangular lumen; columnar epithelial cells were disposed radially; no ventricular appendix and/or intestinal caecum was present. According to morphological features, the nematode was considered belonging to the genus *Anisakis*, but it was not possible to identify it at species level (Fig. 1c).

### Molecular identification of CC1 and CC2 by direct DNA sequencing

**Fig. 2:**
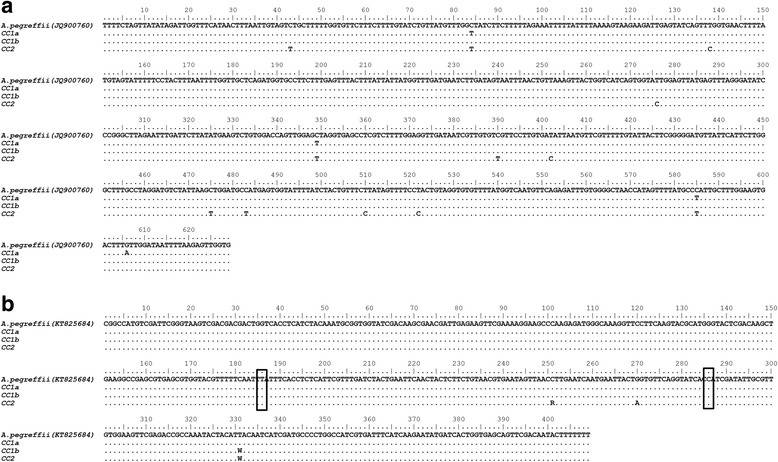
**a** Alignment of the mtDNA *cox2* (629 bp) sequences obtained from two larval nematodes removed from CC1 (**a** and **b**) and from one larva of the CC2, with respect to the sequences previously obtained from a clinical case due to *A. pegreffii* (Genbank accession number JQ900760). The nematode larvae from CC1 and CC2 were identified as *Anisakis pegreffii,* with identity of 99%. The alignment was performed using BioEdit [41]. Dots indicate bps identity. **b** Alignment of EF1 α-1 nDNA (409 bp) sequences of CC1 (**a** and **b**) of two larval nematodes removed from CC1 (**a** and **b**) and from one larva from intestine of CC2, with respect to the sequences previously obtained of *A. pegreffii* (Genbank accession number KT825684) [19]. The nematode larvae from CC1 (**a** and **b**) and CC2 were identified as *Anisakis pegreffii,* with identity of 100 and 99%, respectively. According to the nucleotide diagnostic positions i.e. showing a T and a C in *A. pegreffii*, respectively, at position 186, and 286, the nematode larvae from the CC1 and CC2 were identified as *Anisakis pegreffii*

The mtDNA *cox2* sequences obtained from the larval nematodes removed from the stomach in CC1 and the larva recovered in the intestine of CC2, showed 99 or 100% similarity with sequences of mtDNA *cox-2* previously obtained for the species *A. pegreffii* [18] and retrievable from GenBank. These mtDNA *cox2* sequences isolated from CC1 and CC2 were deposited in GenBank under the accession numbers: KY073403 e KY073404 (Fig. 2a).

In addition, according to the diagnostic positions found at the EF1 α − 1 region of the nDNA (409 bp), i.e. showing a T and a C in *A. pegreffii*, respectively, at position 186, and 286 the same larval specimens were assigned to the species *A. pegreffii* (Fig. 2b). Sequences of EF1 α − 1 region of nDNA were deposited in GenBank under the accession numbers: KY118808 and KY118809.

### Molecular identification of CC1, CC2 and CC3 by RT-PCR assay

**Fig. 3:**
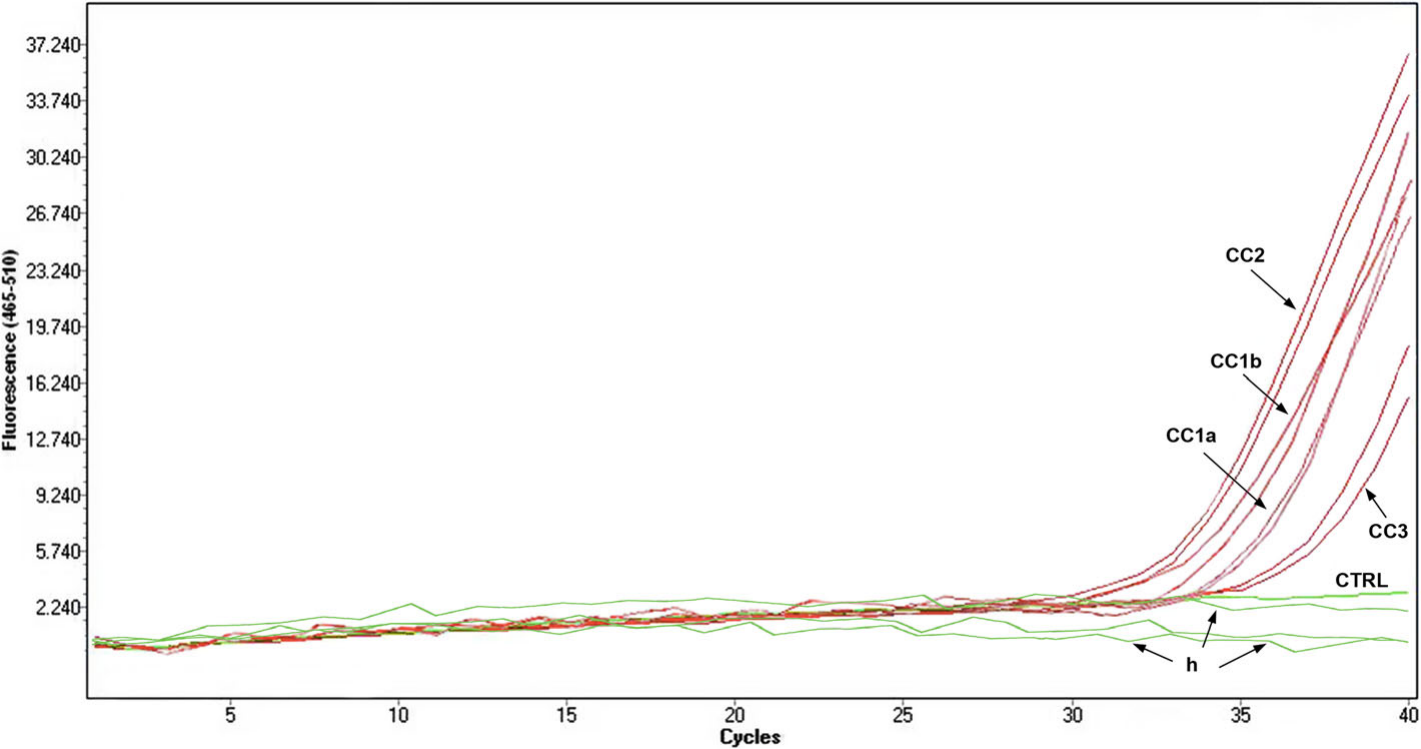
Real Time PCR targeting the DNA of *Anisakis pegreffii* larvae from clinical cases CC1a and CC1b, CC2 and CC3. DNA from human blood sample was also included (*h*). The extracted DNA were tested, in duplicate, for clinical cases and, in triplicate, for the human tissue (*h*); CTRL: negative control

The extremely low amount of extracted DNA (<0,01 ng/μl, Qubit2.0 BR-dDNA kit), did not allow direct sequencing. On the other hand, the RT-PCR primers/probe system procedure, showed a consistent fluorescent signal at 510 nm (FAM dye), as defined for the species *A. pegreffii* [our unpublished data], but showing a relatively high C_t_ values (~34), as in Fig. 3, due to the low amount of DNA.

The RT-PCR assay was also used for the CC1 and CC2 DNA samples. The analysis was performed on CC1, CC2, CC3 in duplicates and, on human tissue (*h*), in triplicates. The limit of detection was established at a value under 0.0006 ng/μl. The reaction efficiency value was *E* = 2.013, with a slope value of σ = −3.29. No cross-reactivity with the genomic DNA from human blood sample was observed.

### Immunoblotting assay

**Fig. 4:**
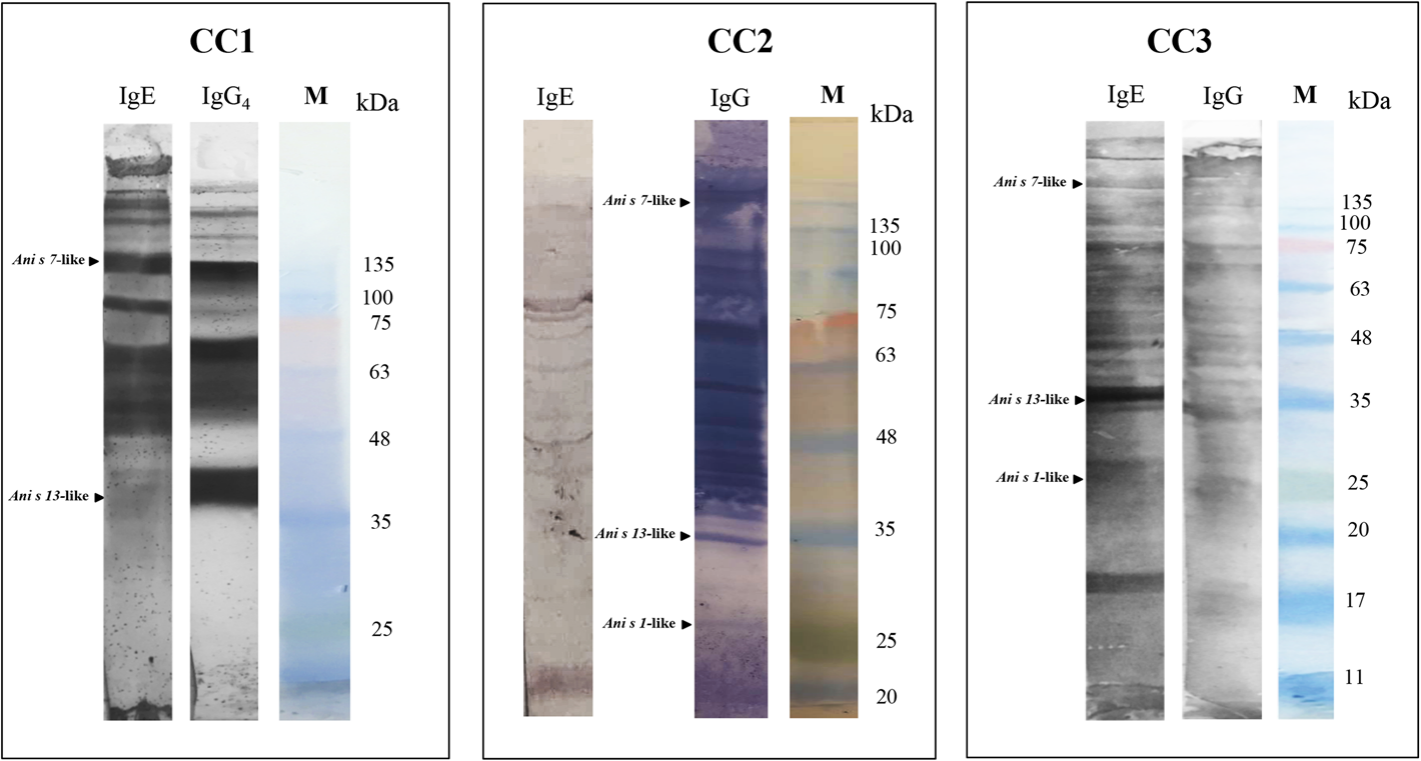
Immunoblotting assay on serum samples from clinical cases CC1, CC2 and CC3, by using ES of *Anisakis pegreffii* larvae. The serum from CC1 shows IgE and IgG_4_ reactivity with bands at 37 kDa (*Ani s 13*-like) and 139 kDa (*Ani s 7*-like). IgG from CC2 reacts with bands at 37 kDa (*Ani s 13*-like), and 139 kDa (*Ani s 7*-like). Finally, the serum from CC3 showed IgE and IgG reactivity bands around 24 kDa (*Ani s 1*-like), 37 kDa (*Ani s 13*-like) and 139 kDa (*Ani s 7*-like). M: marker

Among the three sera samples available in the present study, the serum from CC1 revealed a specific IgE and IgG_4_ antibody reactivity to two antigens whose relative mobility (M_r_) resulted around 37 kDa and 139 kDa, corresponding to *Ani s 13*-like and *Ani s 7*-like, respectively (Fig. 4). Conversely, the serum from CC2 showed IgG reactivity against *Ani s 13*-like and *Ani s 7*-like (Fig. 4). Finally, serum from CC3 showed both IgE and IgG reactivity against proteins/antigens whose M_r._ was corresponding to *Ani s 13*-like*, Ani s 7*-like and with respect to another band recognized by both IgE and IgG, was observed, having a M_r._ of 24 kDa, corresponding to *Ani s 1*-like (Fig. 4).

### Discussion

We documented one gastric and two intestinal cases of invasive anisakiasis caused by the species *A. pegreffii.* In Italy, previous cases of gastric [12, 22–26] and intestinal [24, 27, 28] anisakiasis were reported. However, in those cases, despite the histological findings and morphological features allowed disclosing the presence of a larval *Anisakis* sp., the identification at the species level was not possible. By contrast, in the last decades, the application of molecular tools in this group of parasites has allowed the identification of larvae infecting humans, thus enlarging the knowledge about this fish-borne zoonosis [9–16]. Furthermore, the implementation of the methodologies based on PCR, as those concerning DNA purification and extraction, have allowed to develop a method for the identification of *A. pegreffii* even from the paraffin-embedded tissues of surgically removed granulomas caused by the parasite [11]. This method was recently successfully applied also in an archival case in Croatia [13].

In the present study, a more sensitive method for the accurate and rapid identification of the etiological agent of human anisakiasis, based on RT-PCR hydrolisis probe, was utilized. This method detected up to 0.0006 ng/μl of DNA of the parasite. The RT-PCR probe assay was assessed for the first time, on a human tissue, (eosinophilic granuloma) whose etiological agent would have been undetectable as inferred by direct DNA sequencing, due to the low quantity of DNA available. This situation could also occur when bioptic tissues are collected by endoscopy from ulcers caused by the parasite in the gastric and/or intestinal mucosa, without direct observation of the parasite itself. Indeed, since the symptoms of anisakiasias are not pathognomonic, the acute form could be misdiagnosed. As a consequence, the infection could become chronic, leading to granuloma formation. Therefore, the early removal and identification of the worm makes the best prevention of formation of eosinophilic granuloma caused by the allergic reaction to the degenerated larva in the chronic infection.

The possibility to use sensitive and specific methods for the molecular diagnosis of human anisakiasis, such as a RT-PCR, as here proposed, will allow to enlarge the knowledge about its occurrence in human populations that regularly consume raw or undercooked fish.

In this respect, in Italy the consumption of home-made raw marinated anchovies that were previously not exposed to rapid freezing (−20 °C for 24 h), as compulsory by European Community regulation (EC no 1276/2011), represents the main risk to contract this fish-borne zoonosis [12]. Indeed, also in the cases, here described, the source of the infection was “home-made raw marinated anchovies”. On the other hand, anchovies *Engraulis encrasicolus*, is one the most important fish resource in the Mediterranean countries; this species was found to be heavily infected by *Anisakis s*pp. larvae, even in its flesh (i.e. edible parts), in some fishing grounds of the Mediterranean Sea [29, 30]. In addition, it was demonstrated that a migration of *A. pegreffii* larvae from the viscera to the edible parts of the *E. encrasicolus* occurs *post-mortem*, with temperature- and time-dependent larval motility [29]. It is therefore important to increase the control of the temperature of the fish storage after landing, up to the domestic level.

*A. pegreffii* is not the only species able to cause human anisakiasis. In Japan, Umehara et al. [14] and Arai et al. [15] recognized *A. simplex* (s .s.) as the main etiological agent of anisakiasis in a large number of patients. The opposite situation was found in South Korea, where Lim et al. [16] described the infection in several patients as caused by *A. pegreffii*, but in one patient was due to *A. simplex* (s. s.)*,* However, those findings could be related both to the fishing ground of the source of the infection (i. e. the infected fish consumed), and to the geographical distribution of the two sibling species of *Anisakis*. Indeed, while the Japanese waters are a sympatric area of the two species from South Pacific area, so far the species *A. simplex* (s. s.) has not been recorded in fish hosts; by contrast, in the same geographical area, *A. pegreffii* is present, also in co-infection with *A. berlandi*, in several fish and cetacean hosts [7, 8, 18].

Concerning the pathogenic potential of *A. pegreffii* in comparison with *A. simplex* (s. s.)*,* according to the literature, *A. pegreffii* has been identified at molecular level in several cases of Gastro Allergic Anisakiasis (GAA) in Italy, characterized by the occurrence of urticaria manifestation and/or edema of oral mucosae [12]. Conversely, so far, no data have been recorded about the GAA due to *A. simplex* (s. s.), identified in clinical cases by molecular means [31]. Of course, this does not exclude the possibility that also *A. simplex* (s. s.) is able to elicit that immune response in humans causing GAA, also in consideration that some authors are reporting a higher capacity of this species to survive the pH condition of human stomach [32, 33] and to penetrate at higher percentage in the muscle of natural fish host [34, 35], but also in its accidental host (humans) [9–16]. However, in the clinical cases here described, in spite of the fact that their etiological agent was *A. pegreffii*, we did not notice any allergic reaction; this finding is similar to that found by Lim et al. [16].

In the present study, we have also performed Immunoblotting assay to study the *Anisakis*-specific immune response in three different cases of anisakiasis due to *A. pegreffii*. Interestingly, the serum from the patient suffering of the eosinophilic granuloma (CC3) showed immunoreactivity at the Immunoblotting assay, at both IgE and IgG against the three most frequent antigens indicative of the infection by *A. pegreffii* (i.e. *Ani s 1*-like, *Ani s 7*-like and *Ani s 13*-like). Whereas, the sera from patient CC1 and CC2 did not react with antigen around *Ani s 1*-like*.* Hypotheses to explain these last findings could be related to: *i)* the serum available from the CC1 was taken just during patient’s hospitalization (i.e. few days after the infected fish intake); on the other hand, it has been reported that IgE reaction against *Ani s 1* increased after 1 month from the infection [2, 36, 37]; *ii)* in both CC1 and CC2 cases, the parasite larva was found, at least, during the first 7 days after the infection*.*

Interestingly, the only gastric case of anisakiasis (i.e. CC1), here studied, showed IgG_4_ reactivity against *Ani s 7*-like. Typical for a parasite-induced immunologic reaction, previous studies observed the concomitant production of both IgE and other immunoglobulin isotypes, such as IgG_4_, which are mediated by the same Th-2 mechanism [38–40]. Further, IgG_4_ response to *Ani s 7* has been suggested as a marker of gastric allergic anisakiasis due to *A. simplex* [40]. Thus, the IgG4 seropositivity against *Ani s 7-*like observed in patient CC1 seems to be in accordance with previous observation [40].

Finally, a band of immunoreactivity having a relative mobility (Mr) around 37 kDa, likely corresponding to *Ani s 13*-like [21]*,* was observed in all the sera tested in the present study; the same antigen was found in all the sera from patients in a large survey carried out on IgE sensitization by *A. pegreffii,* in Italy [21]. This suggests that *Ani s 13*-like could be a good candidate in the diagnosis of human anisakiasis.

### Conclusion

The Real-time PCR primers/probe system, a more sensitive method than direct DNA sequencing for the accurate and rapid identification of etiological agent of human anisakiasis, was successfully assessed, for the first time, in the cases here presented. Overall, these observations highlight the importance of the application of molecular tools, such as RT-PCR, in the identification of the etiological agent of human anisakiasis, especially when low DNA quantity is available. Further, this study reinforces the need to use both molecular tools and immunodiagnosis - possibly by Immunoblotting assay and/or ELISA tests - in clinical cases of human anisakiasis, occurring worldwide. A multi-method approach will also contribute to increase our knowledge about the pathological findings and immune response related to the infection by different zoonotic species of the genus *Anisakis.*

## Abbreviations

EF1 α − 1: Elongation factor gene
L3: Third stage larvae
M_r._: Relative mobility
mtDNA *cox2*: Mitochondrial DNA cytochrome c oxidase subunit II gene
mtDNA: Mitochondrial DNA
nDNA: Nuclear DNA
RT-PCR: Real-time PCR
